# Co-expression of High-mobility group box 1 protein (HMGB1) and receptor for advanced glycation end products (RAGE) in the prognosis of esophageal squamous cell carcinoma

**DOI:** 10.1007/s12672-022-00527-9

**Published:** 2022-07-13

**Authors:** Lingzhao Li, Narasimha M. Beeraka, Linsen Xie, Li Dong, Junqi Liu, Lei Wang

**Affiliations:** 1 Department of Clinical Laboratory, Zhengzhou Central Hospital Affiliated to Zhengzhou University, Zhengzhou, 450007 Henan People’s Republic of China; 2 Department of Radiation Oncology, The First Affiliated Hospital of Zhengzhou University, Zhengzhou, 450000 Henan People’s Republic of China; 3 Department of Radiation Oncology, Zhengzhou Central Hospital Affiliated to Zhengzhou University, 195# Tongbai Road, Zhengzhou, 450052 Henan People’s Republic of China; 4 Department of Human Anatomy, I.M. Sechenov First Moscow State Medical University (Sechenov University), 8/2 Trubetskaya Street, Moscow, 119991 Russian Federation

**Keywords:** Esophageal squamous cell carcinoma (ESCC), HMGB1, RAGE, Clinicopathological findings, Overall survival

## Abstract

Esophageal cancer is a malignant type of cancer with a high mortality rate. The aim of this study is to determine co-expression patterns of High-mobility group box 1 protein (HMGB1) and receptor for advanced glycation end products (RAGE) in ESCC (esophageal squamous cell carcinoma) conditions and their prognostic role in cancer progression. The expression of HMGB1 and RAGE in ESCC tissues has been analyzed using qRT–PCR and Western blotting. Co-localized expression patterns of HMGB1 and RAGE in ESCC tissues were determined using immunohistochemistry and analyzed for clinical-pathological parameters. Overall survival was performed based on co-expression of HMGB1 and RAGE proteins. A higher expression pattern of HMGB1, and RAGE was observed at mRNA and protein level in the ESCC group compared to the adjacent tissue group. Expression of HMGB1 was significantly correlated with lymph node, metastasis, lymphatic invasion, and venous invasion (p < 0.05). RAGE expression exhibited a significant correlation with venous invasion. Overall survival was significantly shorter (P < 0.05) in the patients with co-expression of HMGB1 and RAGE compared to the patients without co-expression. A significant difference in the overall survival was evident between the patients with co-expression of HMGB1 and RAGE and the patients without coexpression. HMGB1 and RAGE expression patterns were associated with aggressive metastatic characteristics of ESCC. The co-expression of HMGB1 and RAGE was correlated with shorter survival times. Results concluded the co-expression patterns of HMGB1 and RAGE exhibited a prognostic relevance in ESCC conditions.

## Introduction

Esophageal cancer is a malignant cancer with a global incidence of 6.5 per 100,000 accompanied by a mortality rate of 3.8 per 100,000 [1, 2]. The incidence of esophageal cancer is predominantly observed in China, Europe, and Africa and a few Asian nations [3, 4]. Nitrosamines, viral infections, genetic susceptibility, dietary habits, and chronic physical stimuli and chronic inflammation contribute to the incidence of esophageal cancer [5]. The inflammatory factors can stimulate the release of oncogenic proteins, and provoke the tumor development across the esophagus. Meanwhile, the extensive levels of cytokines and chemokines in the microenvironment can induce the proliferation of tumor cells across the tumor microenvironment [6].

High-mobility group box 1 protein (HMGB1) or amphoterin is a conservative nuclear protein with a molecular weight of 215 amino acids secreted by monocytes, macrophages and dendritic cells [7]. It is widely distributed across the lymphatic tissue, brain, lung, heart, kidney, and other tissues; majority of HMGB1 proteins could be observed inside the nucleus and it is a non-histone DNA binding protein, and promotes the synthesis of nuclear protein complex involved in the regulation of DNA recombination, replication, repair and transcription process [8]. Similar to cytokines, HMGB1 plays an important role in the cancer progression and also induces immune tolerance in the tumor microenvironment by chronic inflammation [9]. Overexpression of HMGB1 is related to the growth, invasion and metastasis of multiple malignancies including gastric cancer [10], pancreatic cancer [11], colorectal cancer [12], and lung cancer [13].

The receptor for advanced glycation end products (RAGE) is multi-ligands receptor and conducive to the tumor progression in gastric cancer, and pancreatic cancer [10, 14]. The expression of RAGE is highly observed across the lungs, kidneys, brain, liver, and esophagus [15, 16] and it can bind to the different ligands including HMGB1. RAGE and HMGB1 interaction can induce the activation of cell signaling pathways including NF-kB, p38, p44/42 MAPKs involved in the tumor progression and metastasis. It has been reported that the RAGE expression was negatively associated with the tumor invasion and prognosis in ESCC [17]. Furthermore, HMGB1/RAGE expression may have significant implications in the development of ESCC. However, it is unclear that the co-expression of HMGB1 and RAGE exhibits any significant implications pertinent to the prognosis of ESCC. In this study, we aimed to investigate the expression of HMGB1 and RAGE in the ESCC specimens and whether the co-expression of HMGB1 and RAGE is involved in the development and prognosis of ESCC.

## Materials and methods

### Patient data collection

A total of 80 patients with thoracic ESCC (59 males and 21 females) registered at The Zhengzhou Central Hospital of Zhengzhou University during the period of January 2015 and April 2016, were included in this study. For ESCC patients with distant metastasis, the preoperative neoadjuvant chemotherapy was prescribed before the surgery. All the patients underwent esophagectomy and lymph node dissection as a treatment modality against ESCC. Diagnosis was performed after the clinical imaging and biopsy combined with clinical symptoms. According to the guidelines of American Joint Committee on Cancer (AJCC), TNM staging system, the pathological stage of the patient was performed. The clinical data pertinent to every patient such as ‘age, gender, tumor size, tumor grade, stage, and lymph node metastasis’ were obtained. After surgical resection, if the patient exhibited typically higher risk factors (T4a and N1-3, positive margin, vascular cancer thrombectomy), then, adjuvant chemotherapy was given with paclitaxel in combinatorial regimen with cisplatin for 4 cycles. This study was approved by the Ethics Committee of The Zhengzhou Central Hospital of Zhengzhou University. Informed consent was signed by all the patients involved in this study.

### Sample collection

The esophageal tissues were obtained from 80 patients who underwent surgical resection of the esophageal cancer tissue. The specimens were obtained within 30 min of surgical resection. The normal esophageal tissue specimens adjacent to the cancer tissue specifically 5 cm away from the edge of tumor tissue were also collected during the surgery and confirmed pathologically. Tissue fixation was performed using 10% formalin for the immunohistochemistry (IHC) while the other specimens were transferred to − 80 °C refrigerator with liquid nitrogen for storage.

### Immunohistochemistry

The paraffin-embedded tissue blocks were excised into sections with thickness of 4 μm. Hematoxylin and eosin staining (HE) was executed on each section prior to performing IHC. Then, the sections were immunostained by anti-HMGB1 (Abcam, USA) and anti-RAGE (Abcam, USA). In brief, the sections were dewaxed using xylene for two times, for 20 min; later, these sections were subjected to dehydration with the aid of graded alcohol; subsequently the antigen retrieval was executed. Slices were immersed into antigen retrieval solution, and subjected to heating in a microwave oven at 100 °C, and subsequently heated at low temperature for 15 min. Later, the slices were subjected to incubation using 3% H_2_O_2_ for fifteen minutes in order to impair the endogenous antigen. Again, the slices were subjected to the incubation with anti-HMGB1 (Abcam, USA) at a dilution of 1:300, anti-RAGE (Abcam, USA) at a dilution of 1:200. Sections were incubated along with immunohistochemical reagents and washing was performed using PBS for three times. Visualization of reaction was done with the aid of 3,3′-diaminobenzidine (DAB). The sections were stained with hematoxylin for 5 min and the slides examined by two independent pathologists (Dr. quanwu Zhang and Dr. huili Bai, First Affiliated Hospital of Zhengzhou University).

To determine the expression of HMGB1 and RAGE, we selected 10 fields and evaluated the expression in the tumor cells. Subjective estimation of IHC scores was performed according to the staining-intensity. The results were graded on a semiquantitative scale based on the intensity patterns—0: no expression; 1: weak expression; 2: moderate expression; 3: strong expression. For statistical analysis, the negative expression was defined as the intensity patterns with no expression and weak expression, whereas the positive expression was defined as moderate and strong intensity patterns.

### Real-time PCR

Trizol method was used to extract the total RNA from the isolated esophageal tissues (Invitrogen, USA) as per the manufacturer instructions. RNA integrity was evaluated by ethidium bromide nucleic acid staining prior to the agarose gel electrophoresis. According to the manufacturer’s protocols, RNA samples were subjected to the reverse transcription with the aid of High Capacity cDNA Reverse Transcription Kit (Applied Biosystems, USA). Ultra SYBR Mixture (with ROX) (CWBIO, China) was applied for quantitative RT–PCR on an Applied Biosystems 7500 (ABI, USA). The primers used in this work were given in Table 1. Reaction cycling conditions: denaturation was performed for 10 min at 95 °C, followed by 15 s (35 cycles) at 95 °C, and 1 min at 60 °C. All samples were tested in triplicates to ensure reproducibility. The relative fold changes in gene expression of HMGB1 and RAGE were ascertained by the 2^− ΔΔCT^ method in the two groups.

**Table 1:** Primers used for qRT–PCR quantifications

Gene name	Direction	Primer sequence
HMGB1	Forward	GTTCAAGGATCCCAATGCAC
	Reverse	GATTTTTGGGCGATACTCAGA
RAGE	Forward	AGAAACCGGTGATGAAGGAC
	Reverse	TCGAGTCTGGGTTGTCGTTT
GAPDH	Forward	AGCCACATCGCTCAGACA
	Reverse	GCCCAATACGACCAAATCC

### Western blotting

Extraction of the total protein was performed from the frozen tissues with the aid of lysis buffer (Solarbio, China). BCA assay was used to measure the protein concentration (Sangon, China) according to the manufacturers’ protocol. Separation of total protein samples was executed using 10% SDS polyacrylamide gel electrophoresis, and then transferred onto the PVDF (Millipore, USA). Later, the transmembrane was subjected to blocking using a blocking buffer made of ‘5% nonfat milk, 0.1% Tween 20’. Later, the membranes were probed with anti-HMGB1 (1:1000 dilution, Abcam, USA), anti-RAGE (1:2000 dilution, Abcam, USA) primary antibodies and incubated overnight at 4 °C, respectively. Next, the membranes were washed 3 times using PBS. Then, the membranes were subjected to incubation for two hours at room temperature with secondary antibodies (1:2000 dilution, Odyssey CIX, USA). The blots were observed using Infrared Laser Scanning Imaging System (Odyssey CIX, USA).

### Statistical analysis

SPSS 22.0 was used for performing the statistical analyses. The *χ*^2^ test were used to analyze the group differences in IHC procedures. The differences in mRNA and protein expression were performed by deciphering through paired-sample *t* tests between two groups. The Fisher’s exact tests and *χ*^2^ test were used to ascertain the relationship between HMGB1 expression and clinical-pathological characteristics. Univariate analysis and multivariate survival analyses were performed using a Cox proportional hazard model in order to identify the independent factors significantly related to patient prognosis. Overall survival analysis was prepared using Kaplan–Meier method whereas the log-rank test was executed in order to compare the survival curves. *P* < 0.05 was considered to be statistically significant.

## Results

### HMGB1 and RAGE overexpression patterns in ESCC

**Fig. 1:**
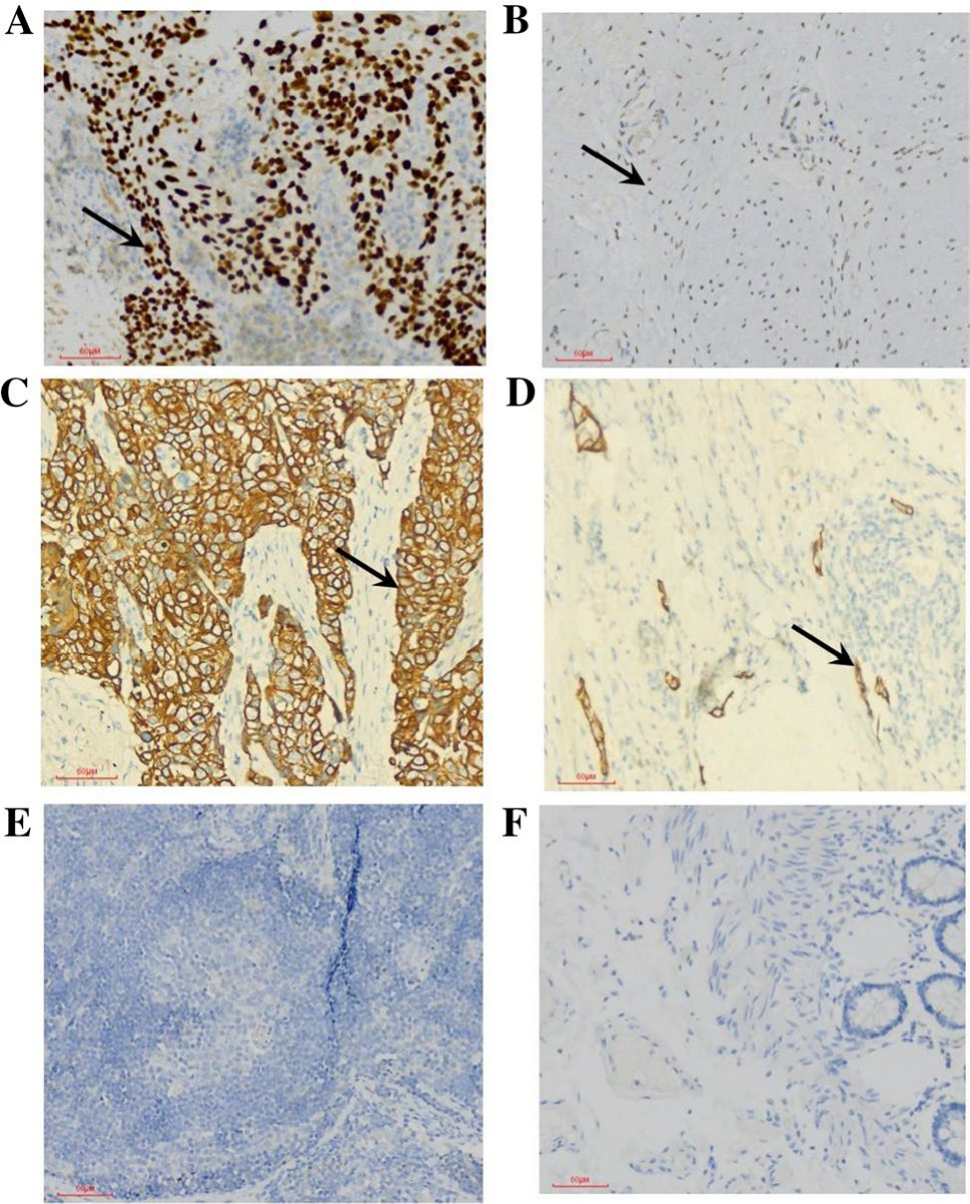
IHC: Comparative HMGB1 and RAGE expression patterns in the tumor esophageal tissue (ESCC) and adjacent esophageal tissue. **A** Positive staining for HMGB1 in the ESCC group. **B** Positive staining for HMGB1 in the adjacent tissue group. **C** Positive staining for RAGE in the ESCC group. **D** Positive staining for RAGE in the adjacent tissue group. **E**, **F** Negative control of two groups. The arrow points to the positive cells. Magnification: 200x

IHC was performed for the isolated esophageal tissues pertinent to the two groups for estimating the localized expression of HMGB1 and RAGE. The HMGB1 protein expression was substantially observed across cytoplasm and nuclei (Fig. 1). Meanwhile, the expression of RAGE was observed in cytoplasm and cell membrane (Fig. 1). HMGB1 and RAGE revealed typically stronger staining intensity patterns in the esophageal tumor tissues of the ESCC group than the adjacent tissue group. Positive expression of HMGB1 was detected in 70% (56/80) in the ESCC group while 47.5% (38/80) in the adjacent tissue group respectively. The positive rate of RAGE expression was 55% (44/80) in the ESCC group, and 36.25% (29/80) in the adjacent tissue group respectively. The detailed data pertinent to the expression patterns of HMGB1 and RAGE were given in Table 2.

**Table 2:** Immunohistochemical analysis of HMGB1 and RAGE expression patterns in two groups including ESCC group and adjacent esophageal tissue group

Immuno staining		No staining	Weak staining	Moderate staining	Strong staining	*χ*^2^	*P* Value
HMGB1	Adjacent tissue	19 (23.7%)	23 (28.7%)	25 (31.2%)	13 (16.2%)	8.36	< 0.01
	ESCC	8 (10%)	16 (20%)	30 (37.5%)	26 (32.5%)		
RAGE	Adjacent tissue	19 (23.7%)	32 (40%)	17 (21.2%)	12 (15%)	5.67	0.017
	ESCC	10 (12.5%)	26 (32.5%)	21 (26.5%)	23 (28.5%)		

*ESCC* Esophageal squamous cell carcinoma

### HMGB1 and RAGE mRNA expression in esophageal tissues of two groups

**Fig. 2:**
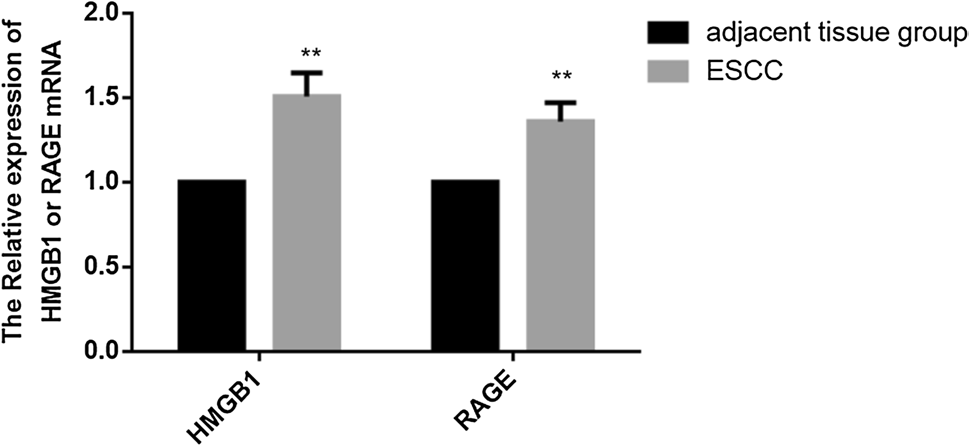
qRT–PCR: The mRNA expression patterns of HMGB1 and RAGE in the tumor esophageal tissue (ESCC) and adjacent esophageal tissue. The mRNA expression of HMGB1 and RAGE were markedly increased in the ESCC group than the control group. Data was presented in mean ± SD, **P < 0.01

The mRNA expression related to HMGB1 and RAGE proteins in the two groups was deciphered by qRT–PCR. The mRNA expression patterns of HMGB1, RAGE were significantly (*P* < 0.01) enhanced across the ESCC tissues than the adjacent tissues (Fig. 2).

### HMGB1 and RAGE proteins expression in esophageal tissues of two groups

**Fig. 3:**
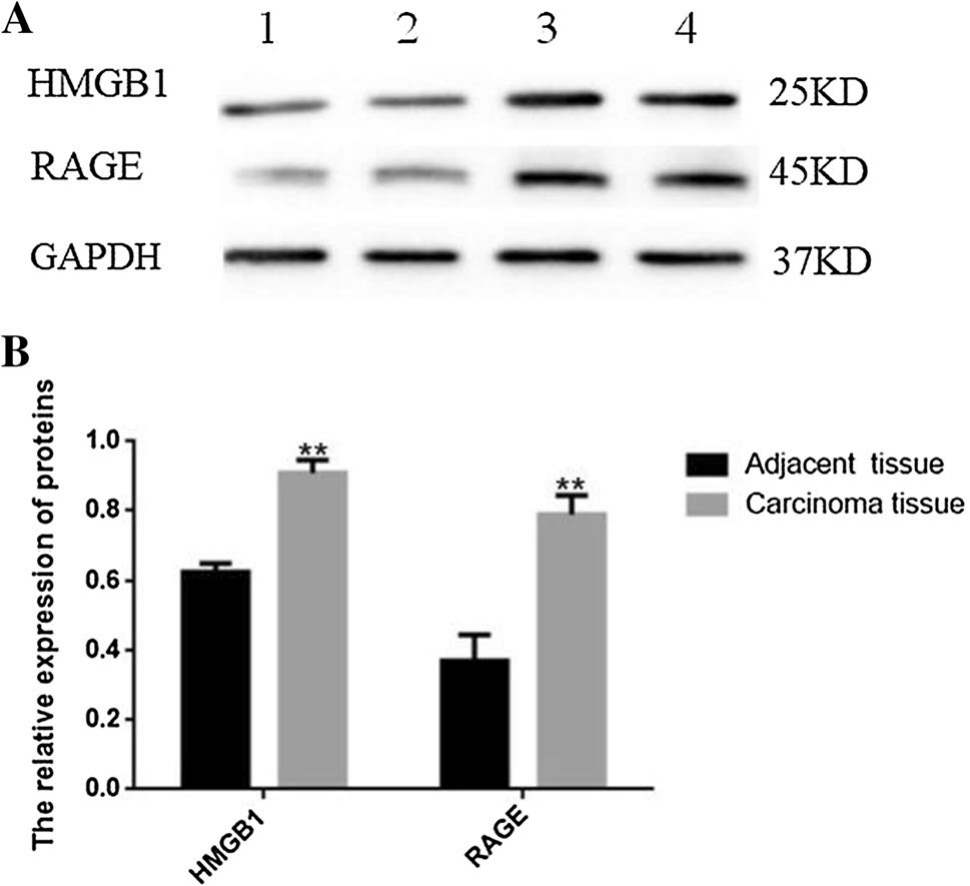
The protein expression patterns of HMGB1 and RAGE in the tumor esophageal tissue (ESCC) and adjacent esophageal tissue. **A** 1, and 2: Banding pattern of HMGB1 and RAGE pertinent to the adjacent esophageal tissue group whereas 3 and 4: banding patterns of HMGB1 and RAGE across the esophageal tissue in ESCC group. **B** Densitometry quantification of HMGB1 and RAGE expression in two groups. The HMGB1 and RAGE protein expression were significantly increased in the ESCC group than the control group. Data were presented as mean ± SD, **P < 0.01

When compared to the adjacent esophageal tissue group, the expression of HMGB1 and RAGE proteins were markedly increased in ESCC group (0.628 ± 0.025 vs. 0.908 ± 0.038, *P* < 0.001; 0.372 ± 0.074 vs. 0.789 ± 0.055, *P* < 0.001; respectively). GAPDH (glyceraldehyde-3-phosphate dehydrogenase) were used as internal control (Fig. 3).

### Expression of HMGB1 and RAGE and clinicopathological correlation

HMGB1 expression was significantly correlated to lymph node status, metastasis status, and T stage (TNM), lymphatic invasion, venous invasion (Table 3). The tumors with positive HMGB1 expression showed typically a higher venous invasion than the tumors with negative HMGB1 expression (*P* < 0.05). The tumors with negative HMGB1 expression showed minimal lymphatic invasion compared to the tumors with positive expression (*P* = 0.011). Besides, a significant correlation between RAGE expression with venous invasion (*P* < 0.05) was observed. However, age, sex, tumor size, histology of tumor, node was not correlated to the expression of RAGE (Table 4). In Table 5, we summarized the relationship between co-expression of HMGB1 and RAGE with clinicopathological characteristics. There was no significant association between co-expression and age, gender, tumor size, histology of tumor in ESCC. However, combined HMGB1/RAGE positive expression was significantly correlated with the TNM stage, lymphatic invasion, and venous invasion (*P* < 0.05).

**Table 3:** Correlation between HMGB1 expression and clinicopatholological characteristics of the patients

Characteristic	Total	HMGB1		*P* Value
	(n = 80)	Positive n = 56 (70%)	Negative n = 24 (30%)	
Sex				0.698
Male	59	42 (75%)	17 (70.8%)	
Female	21	14 (25%)	7 (29.2%)	
Age (years)				0.543
Mean	58.4	58.8	57.9	
Standard deviation	7.9	8.4	8.1	
Tumor size (cm)				0.881
> 5 cm	31	22 (39.3%)	9 (37.5%)	
≤ 5 cm	49	34 (60.7%)	15 (62.5%)	
pT				0.007*
T_1,_ T_2_	35	19 (33.9%)	16 (66.7%)	
T_3,_ T_4_	45	37 (66.1%)	8 (33.3%)	
pN				0.029*
N_0_	26	14 (25%)	12 (50%)	
N_1_	54	42 (75%)	12 (50%)	
pM				0.019*
M_0_	28	15 (26.8%)	13 (54.2%)	
M_1_	52	41 (73.2%)	11 (45.8%)	
Histology				0.308
Well	18	11 (19.6%)	7 (29.2%)	
Moderate	37	27 (48.2%)	10 (41.6%)	
Poor	25	18 (32.2%)	7 (29.2%)	
Lymphatic invasion				0.028*
Negative	32	18 (32.1%)	14 (58.3%)	
Positive	48	38 (67.9%)	10 (41.7%)	
Venous invasion				0.011*
Negative	36	20 (35.7%)	16 (66.7%)	
Positive	44	36 (64.3%)	8 (33.3%)

**Table 4:** Correlation between RAGE expression and clinicopatholological characteristics of the patients

Characteristic	Total	RAGE		*P* Value
	(n = 80)	Positive n = 44 (55%)	Negative n = 36 (45%)	
Sex				0.458
Male	59	31 (70.5%)	28 (77.8%)	
Female	21	13 (29.5%)	8 (22.2%)	
Age (years)				0.324
Mean	58.4	59.2	57.7	
Standard deviation	7.9	7.2	8.2	
Tumor size (cm)				0.661
> 5 cm	31	18 (40.9%)	13 (36.1%)	
≤ 5 cm	49	26 (59.1%)	23 (63.9%)	
pT				0.141
T_1,_ T_2_	35	16 (36.4%)	19 (52.8%)	
T_3,_ T_4_	45	28 (63.6%)	17 (47.2%)	
pN				0.872
N_0_	26	15 (34.1%)	11 (30.6%)	
N_1_	54	29 (65.9%)	25 (69.4%)	
pM				0.221
M_0_	28	18 (40.9%)	10 (27.8%)	
M_1_	52	26 (59.1%)	26 (72.2%)	
Histology				0.545
Well	18	11 (25%)	7 (19.4%)	
Moderate	37	20 (45.5%)	17 (47.2%)	
Poor	25	13 (29.5%)	12 (33.3%)	
Lymphatic invasion				0.521
Negative	32	19 (43.2%)	13 (36.1%)	
Positive	48	25 (56.8%)	23 (63.9%)	
Venous invasion				0.008*
Negative	36	14 (31.8%)	22 (61.1%)	
Positive	44	30 (68.2%)	14 (38.9%)

**Table 5:** Correlation between co-expression of HMGB1 and RAGE in ESCC expression and clinicopatholological characteristics of the patients

Characteristic	Total	HMGB1 + RAGE		*P* Value
	(n = 80)	Co-expression n = 32 (40%)	Non-coexpression n = 48 (60%)	
Sex				0.468
Male	59	25 (78.1%)	34 (70.8%)	
Female	21	7 (21.9%)	14 (29.2%)	
Age (years)				0.652
Mean	58.4	59.1	57.7	
Standard deviation	7.9	7.4	8.5	
Tumor size (cm)				0.454
> 5 cm	31	14 (43.8%)	17 (35.4%)	
≤ 5 cm	49	18 (56.3%)	31 (64.6%)	
pT				0.021*
T_1,_ T_2_	35	9 (28.1%)	26 (54.2%)	
T_3,_ T_4_	45	23 (71.9%)	22 (45.8%)	
pN				0.03*
N_0_	26	6 (18.8%)	20 (41.7%)	
N_1_	54	26 (81.3%)	28 (58.3%)	
pM				0.013*
M_0_	28	6 (18.8%)	22 (45.8%)	
M_1_	52	26 (81.3%)	26 (54.2%)	
Histology				0.328
Well	18	8 (25%)	10 (20.8%)	
Moderate	37	12 (37.5%)	25 (52.1%)	
Poor	25	12 (37.5%)	13 (27.1%)	
Lymphatic invasion				0.007*
Negative	32	7 (21.9%)	25 (52.1%)	
Positive	48	25 (78.1%)	23 (47.9%)	
Venous invasion				<0.001*
Negative	36	5 (15.6%)	31 (64.6%)	
Positive	44	27 (84.4%)	17 (35.3%)	

### Prognostic relevance of HMGB1 and co-expression of HMGB1/RAGE expression in ESCC patients

During the follow-up period of 5 years, a total of 45 patients died whereas 3 patients were lost to follow-up. Univariate analysis revealed that pM stage, lymph node metastasis, venous invasion, HMGB1 expression, and co-expression of HMGB1 and RAGE were significantly associated with poor survival rates (*P* ≤ 0.05, *P* ≤ 0.001) (Table 6). Multivariate regression analysis indicated that venous invasion, HMGB1 expression, and co-expression of HMGB1 and RAGE were independent prognostic factors (Table 7).

**Table 6:** Cox regression survival analysis of factors predicting survival time of patients with ESCC

Clinicopathological characteristics	Total (n = 80)	Exp (B)	95.0% CI for Exp (B)		*P* Value
			Lower	Upper	
Tumor size (cm)		0.889	0.563	1.406	0.616
> 5 cm	31				
≤ 5 cm	49				
pT		0.654	0.417	1.027	0.065
T_1,_ T_2_	35				
T_3,_ T_4_	45				
pN		0.622	0.385	1.005	0.052
N_0_	26				
N_1_	54				
pM		0.603	0.377	0.962	0.034
M_0_	28				
M_1_	52				
Lymphatic invasion		0.375	0.235	0.599	< 0.001
Negative	32				
Positive	48				
Venous invasion		2.251	1.426	3.554	< 0.001
Negative	36				
Positive	44				
HMGB1 expression		2.553	1.552	4.2	< 0.001
Negative	24				
Positive	56				
RAGE expression		0.699	0.446	1.096	0.119
Negative	36				
Positive	44				
Co-expression of HMGB1 and RAGE		2.722	1.69	4.386	< 0.001
Negative	48				
Positive	32

**Table 7:** Survival analyses by multivariate Cox regression analysis

Independent factor	Overall survival			
	Exp (B)	95% CI for Exp (B)		*P* Value
Venous invasion		Lower	Upper	
Negative/positive	2.026	1.157	3.549	< 0.001
HMGB1 expression				
Negative/positive	2.237	1.251	4.001	0.007
Co-expression of HMGB1 and RAGE				
Negative/positive	2.754	1.700	4.463	0.013

**Fig. 4:**
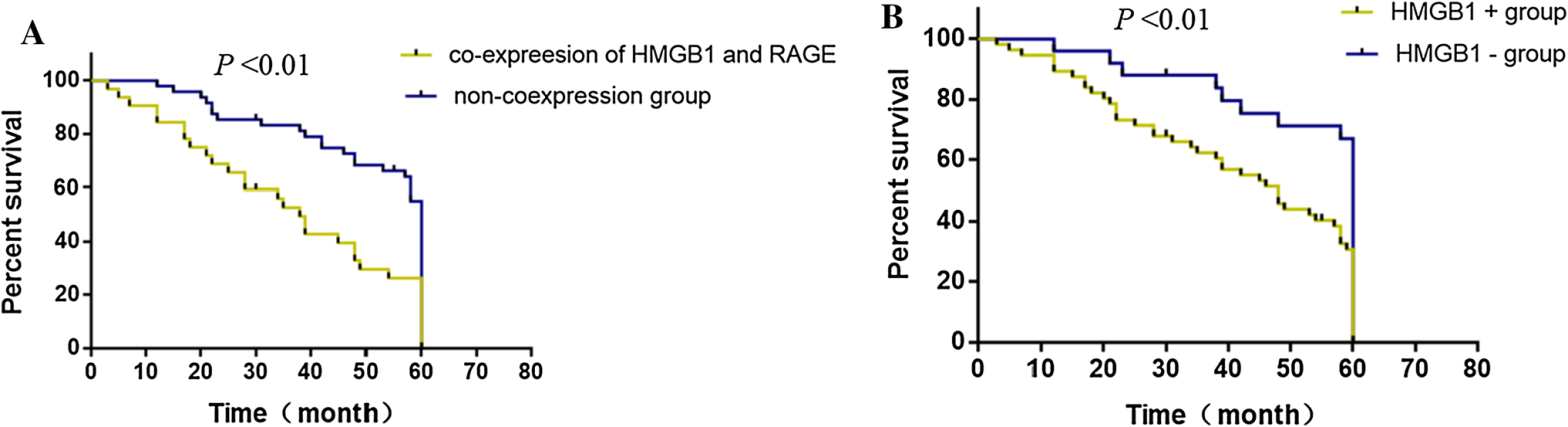
**A** Overall Survival curves depicting the survival rates for the patient group with co-expression of HMGB1 and RAGE when compared to non-expression group pertinent to the ESCC patients. **B** Survival curves for the patients group with HMGB1 ( +) and without HMGB1 (-). *P < 0.01

Among 80 patients, the colocalized expression of HMGB1 and RAGE was observed in 32 patients. Among these 32 patients, overall survival was more than 5 years (25%) for 8 patients. However, the 5-year survival rate of patients without co-expression of HMGB1 and RAGE was 52.1%. The 5-year survival rate of patients with positive HMGB1 expression was 28.6%, whereas the survival rate for patients with negative HMGB1 expression was 66.7%. Survival curves revealed that the patients with combined HMGB1/RAGE positive expression and HMGB1 positive alone reported poor survival rates (Fig. 4). There was a statistically significant difference in overall survival rates between patients with positive and negative expression of HMGB1 and co-expression of HMGB1 and RAGE (*P* < 0.01).

## Discussion

Esophageal malignant tumors can be induced through the chronic inflammatory signaling cascades across the tissues [6]. HMGB1 and RAGE signaling has been playing a crucial role in this process. We analyzed the expression of HMGB1 and RAGE in ESCC samples and adjacent tissues located around the tumor tissue, subsequently ascertained the correlation between the HMGB1 and RAGE expressions and clinicopathologic parameters. This study is the first report describing the prognostic relevance of HMGB1-RAGE in the patients with ESCC.

Substantial expression of RAGE and HMGB1 is evident in the prostate cancer tissues when compared to the expression in untreated or hormone-refractory prostate cancer tissues. In addition, the production of RAGE is typically higher during the metastatic conditions when compared to the nonmetastatic prostate tumors. Thus, HMGB1 expression can contribute to the development, progression of multiple cancers [12, 13, 24–33]. In the present study, the HMGB1 protein was highly expressed in ESCC samples when compared to the adjacent tissues. In addition, a stronger HMGB1 expression was evident in the esophageal tissues of the ESCC group than the adjacent tissue group concluding that our results in line with other reports observed with HMGB1 overexpression in cervical cancer, prostate cancer, and colorectal cancer [18].

A study by Chu-Biao Zhao et al. 2014 reported that a higher expression of RAGE and HMGB1 could induce cancer progression and poor prognosis of prostate cancer [11]. In this study, the IHC of 85 patients with prostate cancer tissues depicted a significant correlation between the HMGB1 and RAGE expression with clinicopathological characteristics and overall survival. Expression of RAGE and HMGB1 are evident in 78.8% and 68.2% patients among 85 cases respectively and the co-expression was observed in the advanced clinical stages and associated with poor overall survival mainly in the patients with stage III and IV [11]. In the present study, HMGB1 expression pattern is positive in 56/80 (70%) patients. There were no correlations observed between HMGB1 expression and sex, age, tumor size or histological type. A significant correlation was observed between HMGB1 expression with node status, metastasis status, and T stage. Chen et al. concluded the significant association of HMGB1 with lymph node metastasis, TNM stage and the prognosis of ESCC patients which was consistent with our findings [19]. A higher expression pattern of HMGB1 was significantly associated with lymphatic invasion and venous invasion. The results showed that overexpression of HMGB1 in ESCC reported to have a significant correlation with the cancer progression.

RAGE is an immunoglobulin superfamily receptor and exhibits its potential role in modulating a variety of diseases. RAGE and its ligand interactions are involved in the inflammation signaling cascades and disease process, including cancer [20]. In this study, the expression of RAGE protein revealed its localization in both cytoplasm and membrane of basal cells in the esophageal epithelium, and the positive expression patterns of RAGE in the ESCC group was significantly higher when compared to the adjacent tissue group. Our study delineated the mRNA and protein expression patterns of HMGB1 in ESCC conditions and demonstrated that the protein expression of HMGB1 in ESCC was significantly greater when compared to the non-cancerous adjacent esophageal tissues. The HMGB1 mRNA expression level is consistent with the protein expression. The higher expression of HMGB1 in the ESCC group indicated that HMGB1 could be associated with aggressive malignant features of ESCC. Our results are in line with previous studies performed in colorectal cancer [21]. Furthermore, there is a vivid enhancement in RAGE expression in the ESCC tissue and these results were consistent with IHC expression patterns of RAGE indicating a strong correlation between the RAGE expression with clinicopathological characteristics of patients. Our results concluded that the positive RAGE expression patterns correlated with venous invasion and these results are in line with reports described by Tateno’s et al. [22]. RAGE expression has profound association with the tumor size, depth of stromal invasion, lymphovascular invasion, and cancer stages in ovarian cancer [23]. However, in our study, the expression of RAGE was independent of the patient's gender, age, tumor size and tumor stage. Overexpression of HMGB1 and RAGE in ESCC indicated a significant association with clinicopathological characteristics.

HMGB1 expression is typically higher in prostate cancer cells undergoing metastasis [34] and its expression exhibited a positive correlation with clinicopathological characteristics among prostate cancer patients concluding its prognostic relevance [35, 36]. A report by Kam et al. [37] reported the implications of cancer cell-generated HMGB1 in the angiogenesis during ESCC progression [37]. Another report by Wenjia Zhang et al. [38] described  the relevance of HMGB1 expression with colorectal cancer development [35]; for instance, a higher HMGB1 in the serum of colorectal cancer patients suggested a significantly higher mRNA and protein expression pertinent to HMGB1 than normal mucosa of colorectal region [35]. Furthermore, the HMGB1 role was described in the bladder urothelial carcinoma as it is involved in fostering malignancy and pathogenesis [36]. HMGB1 expression is typically higher in prostate cancer cells undergoing metastasis [37] and its expression exhibited a positive correlation with clinicopathological characteristics among prostate cancer patients concluding its prognostic relevance [24–33, 36, 37]. Mainly, HMGB1-RAGE signaling has a significant functional role in the development as well as progression of hepatocellular carcinoma; for instance, the expression of HMGB1 is higher in HCC conditions and associated with clinicopathological characteristics. HMGB1-RAGE signaling can modulate the HCC cell cycle [38, 39]. Other repots described the translocation of HMGB1 from nuclei to the cytoplasm during HBV or HCV (hepatitis B virus/ hepatitis V virus) [40–44]. Our results show that co-expression of HMGB1 and RAGE correlates with node status, metastasis status, T stage, lymphatic invasion and venous invasion. HMGB1 overexpression and co-expression of HMGB1 and RAGE could predict clinical prognosis in ESCC. Our results are consistent with Weiling He et al. [45], in which the overexpression of HMGB1 in gastric cancer tissues facilitates the prediction of clinical prognosis [45]. Our results suggest that co-expression of HMGB1 and RAGE contributes to the development and progression of ESCC. Kaplan–Meier survival analysis indicated that the patients with co-expression of HMGB1-RAGE and HMGB1 alone exhibited poor prognoses. Nevertheless, the mechanism by which coexpression of HMGB1 and RAGE promotes the progression of ESCC needs to be further investigated.

## Conclusion

Expression of HMGB1 and RAGE were significantly increased in ESCC patients and their expressions were associated with venous invasion of ESCC; A poor overall survival rate was observed among the patients with the enhanced co-expression of HMGB1 and RAGE in ESCC. Co-expression of HMGB1 and RAGE could be conducive to the progression of ESCC. Co-expression of HMGB1 and RAGE was an independent prognostic factor for the overall survival in patients with ESCC. Patient sample size is a limitation in our study. But, detailed studies with a higher number of patient sample size are required to decipher the clinical prognostic relevance of HMGB1 and RAGE co-expression patterns in ESCC.

## Data Availability

Not applicable.
